# Literature review to assemble the evidence for response scales used in patient-reported outcome measures

**DOI:** 10.1186/s41687-018-0056-3

**Published:** 2018-09-06

**Authors:** Katharine Gries, Pamela Berry, Magdalena Harrington, Mabel Crescioni, Mira Patel, Katja Rudell, Shima Safikhani, Sheryl Pease, Margaret Vernon

**Affiliations:** 1 Janssen Global Services LLC, 700 US 202, Raritan Ave, Raritan, NJ 08869 USA; 2 Shire Pharmaceuticals, 500 Shire Way, Lexington, MA 02421 USA; 3 Critical Path Institute, Patient-Reported Outcome Consortium, 1730 E River Rd, Tucson, AZ 85718 USA; 4 Outcomes & Evidence, Global Health & Value, Pfizer Ltd, Tadworth, Surrey, UK; 5 Evidera, 7101 Wisconsin Ave. Suite 1400, Bethesda, MD 20814 USA; 6 Pfizer Inc., NYC, NY USA

**Keywords:** Patient-reported outcome, Response option, Response scales, Literature review

## Abstract

**Background:**

In the development of patient-reported outcome (PRO) instruments, little documentation is provided on the justification of response scale selection. The selection of response scales is often based on the developers’ preferences or therapeutic area conventions. The purpose of this literature review was to assemble evidence on the selection of response scale types, in PRO instruments. The literature search was conducted in EMBASE, MEDLINE, and PsycINFO databases. Secondary search was conducted on supplementary sources including reference lists of key articles, websites for major PRO-related working groups and consortia, and conference abstracts. Evidence on the selection of verbal rating scale (VRS), numeric rating scale (NRS), and visual analogue scale (VAS) was collated based on pre-determined categories pertinent to the development of PRO instruments: reliability, validity, and responsiveness of PRO instruments, select therapeutic areas, and optimal number of response scale options.

**Results:**

A total of 6713 abstracts were reviewed; 186 full-text references included. There was a lack of consensus in the literature on the justification for response scale type based on the reliability, validity, and responsiveness of a PRO instrument. The type of response scale varied within the following therapeutic areas: asthma, cognition, depression, fatigue in rheumatoid arthritis, and oncology. The optimal number of response options depends on the construct, but quantitative evidence suggests that a 5-point or 6-point VRS was more informative and discriminative than fewer response options.

**Conclusions:**

The VRS, NRS, and VAS are acceptable response scale types in the development of PRO instruments. The empirical evidence on selection of response scales was inconsistent and, therefore, more empirical evidence needs to be generated. In the development of PRO instruments, it is important to consider the measurement properties and therapeutic area and provide justification for the selection of response scale type.

## Background

Response scale selection is a critical aspect in the development of patient-reported outcome (PRO) instruments and has implications for the usability of the measure, the level of precision with which the construct of interest is measured, and the quantitative properties of the outcome score including range, standard deviation, scoring, score interpretation guidelines, and ability of the measure to detect change. Additional complicating factors such as placement of response anchors and exact wording of anchors, cultural comparability/translatability of the format and anchor wording, and ability to migrate the scale to various modes of data collection (paper/pencil, electronic) should be examined when selecting the optimal response scale option for a PRO measure.

Despite the importance of response scale selection for PRO instruments, there is little empirical evidence for the optimal type of response scale and number of response options. For PRO measures with multiple items, 5-point and 7-point verbal rating scales (VRS) are commonly used for adult assessments; examples include the Patient-Reported Outcomes Measurement Information System (PROMIS) item banks and EXAcerbations of Chronic Pulmonary Disease Tool (EXACT®). Eleven-point numeric rating scales (NRS) (particularly recommended for use in pain measurement but used in various other areas as well [1]), and 10 cm (cm) /100 mm (mm) visual analogue scales (VAS) are commonly used for single item adult assessments. In the pediatric literature, there is some evidence that children can reliably distinguish and understand fewer response options than adults. For example, in testing the Childhood Asthma Control Test (cACT), Liu et al. [2] found that a 4-point response scale with no neutral center value was optimal. Furthermore, a graphical scale rather than a NRS or VRS may enhance comprehension of response scales in children [3].

The objective of this literature review was to assemble the evidence on the selection of response scale types to guide the development of PRO instruments. This paper focuses on the overall methodology and results of the literature review. A large body of the available evidence was specific to PRO instruments that were developed for the measure of pain or based on age of the respondent. Because of this, the results of those searches were provided in separate publications [4, 5].

## Methods

A comprehensive review of the scientific literature was conducted to identify response scale types in the development of PRO instruments and the empirical evidence used to justify the appropriate scale type by context of use. The targeted search strategy included formal guidelines or review articles on the selection of response scales and response scale methodology (not specific to PRO instruments) and evidence on the selection of response scales for use in PRO instruments [Table 1]. Evidence was assembled and collated based on pre-determined categories: reliability, validity, and responsiveness of a PRO instrument; select therapeutic areas: asthma, cognition, depression, fatigue in rheumatoid arthritis, and oncology; and the optimal number of response scale options.

**Table 1:** Literature review search terms

No.	Type	Search Terms
Search #1		
#1	Consensus/guideline/ review terms	‘consensus’/exp. OR consensus:ab,ti OR ‘review’/exp. OR review:ab,ti OR ‘practice guideline’/exp. OR guideline*:ab,ti OR ‘expert opinion’:ab,ti NOT ‘institutional review board’
#2	Response scale terms	‘response scale’:ab,ti OR ‘response scales’:ab,ti OR likert:ab,ti OR ‘likert scale’/exp. OR ‘visual analog scale’:ab,ti OR ‘visual analog scales’:ab,ti OR ‘visual analogue scale’:ab,ti OR ‘visual analog scale’/exp. OR ‘numerical rating scale’:ab,ti OR ‘numerical rating scales’:ab,ti OR ‘verbal rating scale’:ab,ti OR ‘verbal rating scales’:ab,ti OR ‘competence scale’:ab,ti OR ‘competence scales’:ab,ti OR ‘frequency scale’:ab,ti OR ‘frequency scales’:ab,ti OR ‘extent scale’:ab,ti OR ‘extent scales’:ab,ti OR ‘comparison scale’:ab,ti OR ‘comparison scales’:ab,ti OR ‘performance scale’:ab,ti OR ‘performance scales’:ab,ti OR ‘developmental scale’:ab,ti OR ‘developmental scales’:ab,ti OR ‘qualitative scale’:ab,ti OR ‘qualitative scales’:ab,ti OR ‘agreement scale’:ab,ti OR ‘agreement scales’:ab,ti OR ‘categorical scale’:ab,ti OR ‘categorical scales’:ab,ti
#3	Selecting terms	select*:ab,ti OR choose:ab,ti OR criteria:ab,ti OR compare:ab,ti OR comparison:ab,ti
#4	Human studies terms	‘animal’/exp. NOT ‘human’/exp.
#5	Clinical trial terms	‘randomized controlled trial’/exp. OR ‘controlled clinical trial’/exp. OR ‘clinical trial’/exp. OR ‘phase 1 clinical trial’/exp. OR ‘phase 2 clinical trial’/exp. OR ‘phase 3 clinical trial’/exp. OR ‘phase 4 clinical trial’/exp. OR ‘multicenter study’/exp. OR random*:ab,ti OR placebo:ab,ti OR trial:ab,ti OR groups:ti OR (singl*:ab,ti OR doubl*:ab,ti OR trebl*:ab,ti OR tripl*:ab,ti AND (mask*:ab,ti OR blind*:ab,ti OR dumm*:ab,ti)) OR ‘double blind procedure’/exp. OR ‘single blind procedure’/exp. OR ‘random allocation’:ab,ti OR ‘open label’:ab,ti OR ‘open labeled’:ab,ti OR ‘open labelled’:ab,ti OR ‘placebo’/exp. OR ‘randomization’/exp. OR ‘crossover procedure’/exp.
#6	Final encompassing terms	#1 AND #2 AND #3 NOT #4 NOT #5 AND ([article]/
Search #2		
#7	Comparison of scales terms	TI ((scale OR measure) N5 (compare* OR merit* OR evaluat* OR consider*)) OR AB ((scale OR measure) N5 (compare* OR merit* OR evaluat* OR consider*))
#8	Merits of scales terms	TI (scor* OR psychometric* OR responsive* OR “cross culture” OR “cross cultural” OR collect* OR “anchor placement” OR “data collection method” OR “internal consistency” OR “test retest” OR construct OR interrater OR standardization OR reliability OR validity OR sensitivity OR specificity OR “item response” OR “intraclass correlation”) OR AB (scor* OR psychometric* OR responsive* OR “cross culture” OR “cross cultural” OR collect* OR “anchor placement” OR “data collection method” OR “internal consistency” OR “test retest” OR construct OR interrater OR standardization OR reliability OR validity OR sensitivity OR specificity OR “item response” OR “intraclass correlation”) OR SU (scor* OR psychometric* OR responsive* OR “cross culture” OR “cross cultural” OR collect* OR “anchor placement” OR “data collection method” OR “internal consistency” OR “test retest” OR construct OR interrater OR standardization OR reliability OR validity OR sensitivity OR specificity OR “item response” OR “intraclass correlation”)
#9	Review/consensus terms	TI (“expert opinion” OR “consensus development”) OR AB (“expert opinion” OR “consensus development”) OR DE “Literature Review”
#10	Final encompassing terms	#2 AND #7 AND #8 NOT #9 NOT #4 NOT #5 AND ([article]/lim OR [article in press]/lim OR [review]/lim) AND [english]/lim AND [2004–2014]/py
Search #3		
#11	PRO terms	‘patient satisfaction’/exp. OR (patient* NEAR/2 satisfaction):ab,ti OR (patient* NEAR/2 reported):ab,ti OR ‘self report’/exp. OR (self NEAR/1 report*):ab,ti OR ‘patient preference’/exp. OR (patient* NEAR/2 preference*):ab,ti OR (patient* NEAR/1 assess*):ab,ti OR ‘self evaluation’:ab,ti OR ‘self evaluations’:ab,ti OR (patient* NEAR/2 rating):ab,ti OR (patient* NEAR/2 rated):ab,ti OR ‘self-completed’:ab,ti OR ‘self-administered’:ab,ti OR (self NEAR/1 assessment*):ab,ti OR ‘self-rated’:ab,ti OR ‘patient based outcome’:ab,ti OR ‘self evaluation’/exp. OR experience*:ab,ti
#12	Format terms	format:ab,ti OR structur*:ab,ti OR ((multiple OR multi OR single OR number) NEAR/4 item*):ab,ti OR (anchor* NEAR/4 (wording OR item*)):ab,ti
#13	Final encompassing terms	#2 AND #11 AND #12 NOT #4 AND ([article]/lim OR [article in press]/lim OR [review]/lim) AND [english]/lim AND [2004–2014]/py
Search #4		
#14	Scoring/ psychometric properties	‘instrumentation’/exp. OR ‘validation study’/exp. OR ‘reproducibility’/exp. OR reproducib*:ab,ti OR ‘psychometrics’ OR psychometr*:ab,ti OR clinimetr*:ab,ti OR clinometr*:ab,ti OR ‘observer variation’/exp. OR observer AND variation:ab,ti OR ‘discriminant analysis’/exp. OR reliab*:ab,ti OR valid*:ab,ti OR coefficient:ab,ti OR ‘internal consistency’:ab,ti OR (cronbach*:ab,ti AND (alpha:ab,ti OR alphas:ab,ti)) OR ‘item correlation’:ab,ti OR ‘item correlations’:ab,ti OR ‘item selection’:ab,ti OR ‘item selections’:ab,ti OR ‘item reduction’:ab,ti OR ‘item reductions’:ab,ti OR agreement OR precision OR imprecision OR ‘precise values’ OR test–retest:ab,ti OR (test:ab,ti AND retest:ab,ti) OR (reliab*:ab,ti AND (test:ab,ti OR retest:ab,ti)) OR stability:ab,ti OR interrater:ab,ti OR ‘inter rater’:ab,ti OR intrarater:ab,ti OR ‘intra rater’:ab,ti OR intertester:ab,ti OR ‘inter tester’:ab,ti OR intratester:ab,ti OR ‘intra tester’:ab,ti OR interobserver:ab,ti OR ‘inter observer’:ab,ti OR intraobserver:ab,ti OR ‘intra observer’:ab,ti OR intertechnician:ab,ti OR intratechnician:ab,ti OR ‘intra technician’:ab,ti OR interexaminer:ab,ti OR ‘inter examiner’:ab,ti OR intraexaminer:ab,ti OR ‘intra examiner’:ab,ti OR interassay:ab,ti OR ‘inter assay’:ab,ti OR intraassay:ab,ti OR ‘intra assay’:ab,ti OR interindividual:ab,ti OR ‘inter individual’:ab,ti OR intraindividual:ab,ti OR ‘intra individual’:ab,ti OR interparticipant:ab,ti OR ‘inter participant’:ab,ti OR intraparticipant:ab,ti OR ‘intra participant’:ab,ti OR kappa:ab,ti OR kappa’s:ab,ti OR kappas:ab,ti OR ‘coefficient of variation’:ab,ti OR repeatab* OR (replicab* OR repeated AND (measure OR measures OR findings OR result OR results OR test OR tests)) OR generaliza*:ab,ti OR generalisa*:ab,ti OR concordance:ab,ti OR (intraclass:ab,ti AND correlation*:ab,ti) OR discriminative:ab,ti OR ‘known group’:ab,ti OR ‘factor analysis’:ab,ti OR ‘factor analyses’:ab,ti OR ‘factor structure’:ab,ti OR ‘factor structures’:ab,ti OR dimensionality:ab,ti OR subscale*:ab,ti OR ‘multitrait scaling analysis’:ab,ti OR ‘multitrait scaling analyses’:ab,ti OR ‘item discriminant’:ab,ti OR ‘interscale correlation’:ab,ti OR ‘interscale correlations’:ab,ti OR (error:ab,ti OR errors:ab,ti AND (measure*:ab,ti OR correlat*:ab,ti OR evaluat*:ab,ti OR accuracy:ab,ti OR accurate:ab,ti OR precision:ab,ti OR mean:ab,ti)) OR ‘individual variability’:ab,ti OR ‘interval variability’:ab,ti OR ‘rate variability’:ab,ti OR ‘variability analysis’:ab,ti OR (uncertainty:ab,ti AND (measurement:ab,ti OR measuring:ab,ti)) OR ‘standard error of measurement’:ab,ti OR sensitiv*:ab,ti OR responsive*:ab,ti OR (limit:ab,ti AND detection:ab,ti) OR ‘minimal detectable concentration’:ab,ti OR interpretab*:ab,ti OR (small*:ab,ti AND (real:ab,ti OR detectable:ab,ti) AND (change:ab,ti OR difference:ab,ti)) OR ‘meaningful change’:ab,ti OR ‘minimal important change’:ab,ti OR ‘minimal important difference’:ab,ti OR ‘minimally important change’:ab,ti OR ‘minimally important difference’:ab,ti OR ‘minimal detectable change’:ab,ti OR ‘minimal detectable difference’:ab,ti OR ‘minimally detectable change’:ab,ti OR ‘minimally detectable difference’:ab,ti OR ‘minimal real change’:ab,ti OR ‘minimal real difference’:ab,ti OR ‘minimally real change’:ab,ti OR ‘minimally real difference’:ab,ti OR ‘ceiling effect’:ab,ti OR ‘floor effect’:ab,ti OR ‘item response model’:ab,ti OR irt:ab,ti OR rasch:ab,ti OR ‘differential item functioning’:ab,ti OR dif:ab,ti OR ‘computer adaptive testing’:ab,ti OR ‘item bank’:ab,ti OR ‘cross-cultural equivalence’:ab,ti
#15	Final encompassing terms	#2 AND #11 AND #3 AND #14 NOT #4 AND ([article]/lim OR [article in press]/lim OR [review]/lim) AND [english]/lim AND [2004–2014]/py
Search #5		
#16	RA (fatigue) terms	‘rheumatoid arthritis’/exp./mj AND ‘fatigue’/exp. OR (‘rheumatoid arthritis’:ab,ti AND fatigue:ab,ti)
#17	Asthma terms	‘asthma’/exp./mj OR asthma:ab,ti
#18	Cognition terms	‘cognition’/exp./mj OR cognition:ab,ti
#19	Depression terms	‘depression’/exp./mj OR depression:ab,ti
#20	SCLC terms	‘lung small cell cancer’/exp./mj OR ‘small cell lung cancer’:ab,ti
#21	Pain terms	‘pain’/exp./mj OR pain:ab,ti
#22	Sub-final terms	#16 OR #17 OR #18 OR #19 OR #20 OR #21
#23	Final encompassing terms	#2 AND #11 AND #3 AND #22 NOT #4 AND ([article]/lim OR [article in press]/lim OR [review]/lim) AND [english]/lim AND [2004–2014]/py

**Fig. 1:**
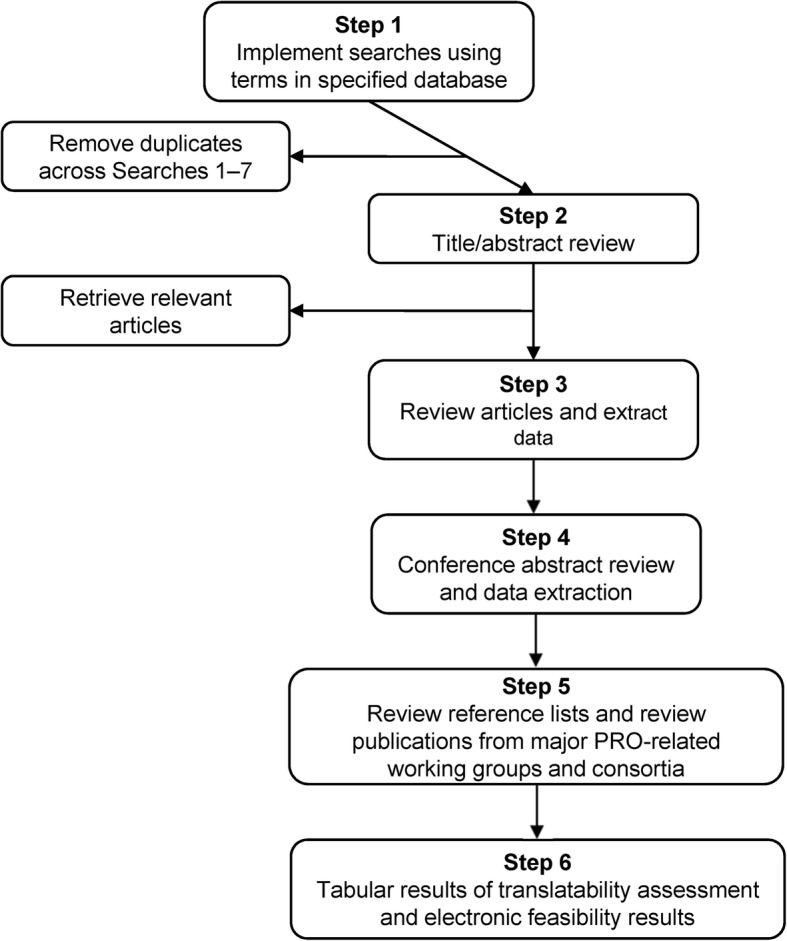
Outline of search procedures and data extraction. PRO: patient-reported outcome

Searches were conducted in the EMBASE, MEDLINE, and PsycINFO databases. Limits were applied to include only articles published in English in the preceding 10 years (2004–2014). The duplicates across individual searches were removed prior to abstract/article review. During the full text article review and data extraction, several supplementary sources were used to identify additional relevant articles for inclusion in the review. These supplementary sources were not limited by publication date, and included the reference lists of key articles, publications not included in the search databases, and websites for major PRO-related working groups and consortia (e.g., PROMIS, NIH Toolbox, Medical Outcomes Study, Neuro-QoL, ASEQ-ME, EORTC, EuroQol Group, and FACIT Measurement System). In addition, conference abstracts were identified and reviewed from annual meetings within the preceding 2 years for Joint Statistical Meetings, Psychometric Society Meetings, International Society for Pharmacoeconomics and Outcomes Research, and International Society for Quality of Life Research. An outline of the review procedure is included in Fig. 1.

### Study selection

During the review process, both abstracts and then full text publications were evaluated for eligibility by two independent reviewers. In the case of non-agreement, a third senior reviewer determined the final judgment. Articles were excluded if they provided no direct or indirect evidence relevant to the search objectives, were not applicable to PRO development, or addressed a therapeutic area not pre-specified for inclusion.

### Synthesis of results

Once articles fitting the search criteria were identified, the relevant data were extracted and summarized. The extraction tables included data on the study objective, study design, study population, therapeutic area, name of PRO instrument, type of response scale, and empirical evidence for response scale selection.

Each article deemed relevant to the review and included in the extraction tables was categorized as including either *direct* evidence or *indirect* evidence. Direct evidence was defined as evidence that provided an answer specific to a research question of interest; for example, direct evidence articles compared empirically the relative robustness or merits of two different response scale types within the same study/population. Indirect evidence was defined as evidence that, while relevant to the review and the overall conclusions, does not directly answer a research question or hypothesis. For example, review articles and articles that evaluated a single response scale type within the study/population (i.e., a study evaluating comprehension of VAS in cognitively impaired patients) were considered to contain indirect evidence.

## Response scale types

The most common types of response scales identified in the literature included: VAS, VRS with or without numerical anchors, NRS, and to a lesser extent graphical scales such as the Faces Scale. Several less commonly used scales were also identified, such as Likert scales and Binary scales.

### Visual analogue scale

The VAS is a scale comprised of a horizontal or vertical line, usually 10 cm (100 mm) in length, anchored at both ends by verbal descriptors [6]. The respondent places a line perpendicular to the VAS line at the point that represents the intensity of the effect in question (e.g., pain). The length of the VAS is imperative on paper, as the score is determined using a ruler and measuring the distance between the lower anchor and the mark made by the respondent (range: 0–100). A variation of the VAS includes either numbers or adjectives indicating intensity along the scale, though this is not encouraged as the numbers and adjectives can bias the results by adding additional components to the scale that may alter interpretation.

### Verbal rating scale

A VRS is a scale that consists of a list of words or phrases describing different levels of the main effect (e.g., pain), in order from least to most intense. The respondent reads the list of verbal descriptors and chooses the one that best describes the intensity of his/her experience [6]. Traditionally a VRS does not contain numbers, but the review identified many examples of VRS with numbers assigned to all or some of the verbal anchors. The study team considered VRS with numbers to be a subcategory of the VRS, with the use of numbers present for scoring purposes and/or to indicate to the respondent that the verbal anchors are meant to have equidistant intervals. Based on the results of the literature review, the VRS was also referred to as a verbal category scale, verbal graphic rating scale, and verbal descriptor scale; and for purposes of this report, were classified as a VRS.

### Numeric rating scale

The NRS is a scale that represents an intensity continuum for respondents to rate the effect (e.g., pain) using a range of integers [6]. The most common NRS is an 11-point scale ranging from 0 (no effect) to 10 (maximal effect). The respondent selects one number that best represents the intensity being experienced. Variations of the NRS included the use of verbal anchors at various points at the middle or ends of a scale; this is common in the context of PRO instrument development.

### Faces scale

A Faces scale is a type of graphical scale that uses photographs or pictures to show a continuum of facial expressions. Line drawings of faces are the most common graphic representation, as their lack of gender or ethnicity indicators makes them applicable to a wider range of respondents [6]. The respondent then selects the face that best describes how he or she is feeling. Verbal labels are usually very simple or non-existent for use in children. The Faces scale does not require reading ability or specific language, thereby facilitating pediatric and multi-cultural comprehension.

### Likert (Likert-type) response scale

The Likert scale is a type of ordinal scale characterized by several features: the scale contains more than one item; response levels are arranged horizontally; response levels are anchored with consecutive integers; response levels are also anchored with verbal labels, which connote more-or-less evenly-spaced gradations; verbal labels are bivalent and symmetrical around a neutral middle; and the scale often measures attitude in terms of level of agreement/disagreement with a target statement [7]. Likert-type scales are most often used to assess agreement, attitude, and probability; while common in social psychology or health psychology scales, they have less use in health outcomes assessments [6]. One exception is a Global Impression of Change scale, where an evaluation of health is made at the start of a new treatment or over a specific time frame. The provision of an odd number of response categories allows respondents to choose a middle, or neutral, response. An even number of response categories forces the respondent to commit themselves to one side of the scale or the other side. The choice between odd and even response categories depends on the desirability of allowing a neutral position. One of the main differences between Likert or Likert-type scales and the VRS is the presence of the neutral middle anchor in the Likert-type scale but not in the VRS, which orders descriptors from least to most measurable attribute(s) [6].

In this literature review, response scales were frequently referred to as Likert or Likert-type; however, most of these scales did not strictly meet the requirements for a Likert scale. Thus, while many scales were referred to as Likert or Likert-type in the original publication, they were more appropriately classified as VRS, and in the literature review will be referred to as VRS.

## Results

### Study selection

The literature search for evidence on types of response scales in formal guidelines or review articles identified 1315 abstracts, plus 13 additional articles selected through secondary sources and 5 conference abstracts. The literature search on the selection of response scale types specific to the development of PRO instruments resulted in 5299 abstracts, 35 abstracts from secondary sources, and 46 conference abstracts. After review the number of references totaled 186 full-text articles. During abstract screening 6199 irrelevant references were excluded, then 463 full text articles were reviewed and 51 conference abstracts. Reasons for exclusion after full-text review included: no discussion or available evidence on the response scale selection (*n* = 233), duplicate (*n* = 36), clinician or observer-rated instrument (*n* = 5), full-text publication not available (n = 3), and 48 conference abstracts were excluded for not containing enough details for data extraction. Results are presented on the selection of response scale types based on reliability, validity, responsiveness, therapeutic areas, and optimal number of response scale options. Over 40% of the included literature (77 references) discussed the selection of response scale type for the measurement of pain and based on study population; therefore, these conclusions were published separately for a comprehensive discussion on the unique issues pertaining to single item pain scales and the differences between pediatric and adult PRO instruments [4, 5].

### Synthesis of results

#### Reliability

Results for the selection of response scale type based on reliability of a PRO instrument were variable. A study on the pediatric population (non-specific therapeutic area) found no difference in test-retest reliability among the VRS, VAS, and a numeric VAS response scale [8]. A study in adults with rheumatoid arthritis found the NRS to be more reliable than VAS or 5-point VRS, with greater test-retest reliability in a subset of participants who were illiterate [9]. Phan and colleagues [10] also found the NRS to have superior test-retest reliability compared to VAS or 4-point VRS when assessed in adults with chronic pruritus. Test-retest reliability was greater for the VAS compared to the other two scale types in healthy adults [11]. Two studies (one on adult geriatric patients with neurological disorders; another on adults with pain) compared 5-point VRS to VAS; VAS was found to have slightly greater test-retest reliability in both studies [12, 13]. A study in adults with angina compared a 5-point VRS to NRS and found no difference in the test-retest reliability of the measure [14]. In another comparison of the NRS and VAS, a study of perceptual voice evaluation in adults for an IVR (interactive voice response) system, there was no difference in intra-rater agreement [15]. However, overall, the NRS and VAS tend to demonstrate better test-retest reliability than the VRS.

#### Validity

Many studies reported concurrence between the response scale types being evaluated within each study. The majority reported large correlations between different items/scales that evaluated the same concept; this is an important consideration in the validity of results compared between response scale types. Only one study in adults with angina reported on the magnitude of correlations using external criterion variables for the response scales under consideration; there was no difference between an NRS and 5-point VRS in concurrent validity [16].

#### Responsiveness

Results for the evaluation of these scale types based on responsiveness, or the ability of the scale to detect change in the underlying condition of a patient with treatment in a naturalistic setting, are provided in Table 2. Results for responsiveness were found only in the pain literature and, as such, may not be generalizable to other therapeutic areas. The comparative responsiveness of VRS and NRS to measure the intensity of pain in patients with chronic pain was assessed directly using two 6-point VRS (current pain) items and four 11-point NRS items from the Brief Pain Inventory (BPI; worst pain, least pain, average pain, and current pain) [17]. The 6-point VRS included the Present Pain Index (PPI) (0 = no pain, 1 = mild, 2 = discomforting, 3 = distressing, 4 = horrible, and 5 = excruciating) and the 6-point Oswestry Disability Index (ODI) (0 = no pain, 1 = very mild, 2 = moderate, 3 = fairly severe, 4 = very severe, and 5 = worst imaginable). For all participants, the standardized response mean (SRM) was small while the VRS-PPI (0.29; 95% CI: 0.17, 0.41) and VRS-ODI (0.27; 95% CI: 0.15, 0.38) were smaller than the BPI NRS measure for current pain (0.36; 95% CI: 0.23, 0.48) [17]. For participants classified as responders, the BPI NRS current pain (0.89, 95% CI: 0.70, 1.07) exhibited large responsiveness and the VRS-PPI (0.58; 95% CI: 0.40, 0.77) and VRS-ODI (0.52; 95% CI: 0.34, 0.70) achieved moderate responsiveness [17].

**Table 2:** Key studies that support response scale selection for PRO instruments based on responsiveness

Reference	Response Scale Type	Methods to Determine Responsiveness^a^	Summary of Results^b^
Grotle et al. 2004 [26]	11-point NRS VAS	SRM	In acute pain, for improved patients NRS SRM = 2.0 and VAS SRM = 1.6. For unchanged patients NRS SRM = 1.0 and VAS SRM = − 0.5. In chronic pain, for improved patients NRS SRM = 1.1 and VAS SRM = 0.4. For unchanged patients NRS SRM = − 0.2 and VAS SRM = 0.1.
Skovlund et al. 2005 [27]	VAS: 100 mm line anchored at no pain/discomfort and pain/discomfort 4-point VRS: none, mild, moderate, severe	Sensitivity of scales with multiple simulations	Cross-sectional analyses with multiple simulations to understand the sensitivity of scales. The VAS consistently gave higher power to detect true differences in pain ratings than the 4-point VRS.
Chanques et al. 2010 [16]	11-point NRS 5-point VRS (no pain, mild pain, moderate pain, severe pain, extreme pain) VAS: 10-cm line anchored at no pain and extreme pain	ES Type of ES (Cohen’s d or SRM) not provided in the reference	Patients identified NRS was the easiest, most accurate and preferred scale in comparison with 5-point VRS and VAS. NRS demonstrated the best sensitivity (96.6%) and negative predictive value (89.6%) whereas VRS demonstrated the best specificity (70.7%) and positive predictive value (86.3%). VAS demonstrated the lowest performance, except for the negative predictive value, which was comparable to VRS ES for 11-point NRS: 1.18 ES for 5-point VRS: 0.94 ES for VAS: 1.13 (vertical orientation)
Dogan et al. 2012 [28]	Faces scale: 7-point horizontal scale that defines feels due to pain. First face represents no pain and the last face represents the worst possible pain VAS: 10-cm horizontal line anchored at no pain and severe pain.	Calculated ES (SRM)	Faces scale ES = 1.78 VAS ES = 1.36
Chien et al. 2013 [17]	11-point NRS (several different BPI scales) 6-point VRS - PPI (no pain, mild, discomforting, distressing, horrible, excruciating) 6-point VRS - ODI (no pain, very mild, moderate, fairly severe, very severe, the worst imaginable)	SRM	Results for all participants: 11-point NRS SRM: ranged from 0.17 to 0.42 6-point VRS SRM: ranged from 0.27 to 0.29
Gonzalez-Fernandez et al. 2014 [29]	VAS (100 mm line) NRS: (gLMS = VAS with the addition of numbers)	Between group difference	The mean (SD) VAS score was 6.13 (2.27) and the mean (SD) NRS score (after scaling to a 0–10 scale) was 4.35 (2.52), with medians of 7 and 4, respectively. The mean difference between the two scores (VAS and NRS) was + 1.78 (*P* < 0.0001).

*PRO* patient-reported outcome, *NRS* numeric rating scale, *VAS* visual analogue scale, *SRM* standardized response mean, *VRS* verbal rating scale, *ES* effect size, *BPI* Brief Pain Inventory, *ODI* Oswestry Disability Index, *gLMS* general Labeled Magnitude Scale, *SD* standard deviation

^a^SRM calculated by dividing the mean change by the standard deviation of the mean change scores. Effect size of 0.2 = small, 0.5 = moderate, and > 0.8 = large clinical change

^b^All references provided direct evidence: Primary research that compares different response scales within study

#### Therapeutic area

Results to support the selection of response scale type based on select therapeutic areas are provided in Table 3. A 5-point VRS used in a PRO instrument evaluating asthma was well understood and acceptable to adults and a 4-point VRS with graphics was understood by children (ages 4 through 11), based on cognitive interviews [2, 18]. Patients with cognitive impairment preferred a VRS over a VAS, but test-retest reliability was similar for both formats [13]. For depression, cognitive interviews supported use of an 11-point NRS, and a 4-point VRS was just as precise in measurements as a 5-point VRS [19]. For fatigue in RA, the VAS and NRS were correlated but not interchangeable; meanwhile, scores from the NRS were higher than the VAS, and patients found the VAS more difficult to understand [20]. Results in oncology studies support use of an 11-point NRS, VAS, VRS, and graphical scales based on the contexts of use and study populations.

**Table 3:** Key studies that support response scale selection used in PRO instruments based on select therapeutic areas

References	Study Type, Evidence Type^a^, Grade^b^	Response Scale Type	Objective	Summary of Results
Asthma				
Sherbourne et al. 2012 [18]	Cross-sectional observational study, Indirect, C	5-point VRS	Develop asthma-specific quality of life items	A 5-point VRS for asthma quality of life assessment in adults was understood based on qualitative research with patients (cognitive interviews).
Liu et al. 2007 [2]	Cross-sectional observational study, Indirect, C	4-point VRS	Develop and validate the Childhood Asthma Control Test (C-ACT)	Children between the ages of 4 and 11 could understand and complete a 4-point VRS assisted by facial graphics.
Cognition				
Hagell and Knutsson 2013 [13]	Prospective, observational study, Direct, A	5-point VRS and VAS	Compare test-retest properties of 2 general health single item response formats among people with neurological disorders	Test-retest reliability assessments were similar for both formats, however patients preferred the VRS over the VAS format.
Depression				
Preston et al. 2011 [19]	Cross-sectional observational study, Direct, A	4-point VRS and 5-point VRS	Evaluate the precision of the 5-point VRS response scale utilized in the emotional distress PROMIS item bank	The 5-point response options are not always equally spaced (i.e., do not meet the assumptions of an equal interval scale) and 4-point response categories were as precise as five.
Lasch et al. 2012 [30]	Cross-sectional observational study, Indirect, C	11-point NRS	Develop a content valid PRO measure for Major Depressive Disorder (MDD)	Cognitive interview demonstrated that an 11-point NRS was well understood and appropriate for evaluating concepts.
Rheumatoid Arthritis (Fatigue)				
Hewlett et al. 2007 [31]	Review, Indirect, B	VAS and NRS	Systematic literature review to identify fatigue in rheumatoid arthritis scales; assess scale measurement properties	A VAS scale was the most frequently utilized scale to evaluate fatigue in rheumatoid arthritis and shows evidence of validity but there was no standardized VAS scale to evaluate fatigue in rheumatoid arthritis as scales were study specific. NRS used to evaluate fatigue in rheumatoid arthritis showed some evidence of construct validity but data on criterion validity, reliability, or sensitivity were not found.
Nicklin et al. 2010 [32]	Cross-sectional observational study, Direct, A	VAS and NRS	Develop and validate a patient reported outcome measure of fatigue in RA, the Bristol RA Fatigue- Multidimensional Questionnaire (BRAF-MDQ) and the Bristol RA Fatigue (BRAF) short scales (VAS/NRS)	The final wording for fatigue severity, effect, and coping VAS/NRS scales was based on focus group recommendations and required measurement properties. The VAS /NRS were understoodby all patients in the way they were intended by the authors. Vertical orientation of the scales enhanced comprehension (rather than horizontal). The NRS and VAS scales were correlated between 0.68–0.78, and showed similar criterion and construct validity. The NRS produced slightly higher scores than the VAS and although the differences were not significant, the results demonstrate the scales are not interchangeable. Although the VAS and NRS performed in similar ways, the NRS was selected for use in evaluating fatigue in this population since some patients found the VAS difficult to understand and because the NRS is easier to score.
Khanna et al. 2008 [33]	Prospective, observational study, Indirect, C	VAS	Evaluate score interpretation (MID) for a fatigue VAS	Mean MID estimates ranged from −0.82 to −1.12 for improvement and 1.13 to 1.26 for worsening (range of 0–10) for a fatigue VAS. These results were similar to those see in RA clinical trials.
Oncology				
Koshy et al. 2004 [34]	Cross-sectional, observational study, Direct, A	VAS, VRS, Graphical rating scales	Determine patient preferences for pain assessment scale type	Most patients (56%) preferred the pain VAS, 30% preferred the graphical (coin) rating scale, 13% preferred the VRS, and no patients preferred the graphical (color) scale. Findings of statistically significant positive correlations between the VAS and VRS suggest both represent similar pain intensity, and both could be used as reliable pain assessment tools. A single item VAS was recommended for evaluating pain in oncology patients because it is reliable and well understood, and preferred by most patients in this study.
Anderson et al. 2007 [35]	Review, Indirect, B	VAS, VRS, and NRS	Review of pain assessment scales for us in an oncology population	Pain intensity ratings using the VAS, NRS, and VRS are highly inter-correlated. The NRS is easily understood by most patients, recommended in many pain treatment guidelines, and may be more reliable than the VAS in clinical trials, particularly with low literacy patients. Pediatric cancer pain scales including color scales, pain thermometers, and Faces scales are suitable for evaluating cancer pain in children under 5 years of age. Children over the age of 5 years can typically complete NRS or VAS.
Rohan 2012 [36]	Review, Indirect, B	VRS and 11-point NRS	Review of distress screening measures used in oncology	A review of the multi-item Hospital Anxiety and Depression Scale (HADS) and the Brief Symptom Inventory- 18 (BSI-18) scale, and a single item Distress Thermometer (11-point NRS) concluded the Distress Thermometer was as discriminative as the multi-item HADS and BSI-18.
Sigurdardottir et al. 2014 [37]	Delphi-process, Indirect, D	NRS	Delphi process to obtain consensus on a basic set of core variables to describe or classify a palliative care cancer population	The 11-point NRS scale was recommended to evaluate important aspects of palliative care in cancer (e.g., appetite, depression, anxiety) and PRO instrument selection should always be undertaken with consideration of specific objectives, samples, treatments, and available resources.
King et al. 2014 [38]	Prospective observational study, Direct, A	11-point NRS and VAS	Determine optimal instrument to measure subjective symptom benefit in clinical trials of palliative	For an ovarian symptom PRO measure, the 11-point NRS was preferable over the VAS and VRS due to improved responsiveness, ease of use, and compliance.
Jacobs et al. 2013 [39]	Prospective observational study, Indirect, C	Faces scale	Psychometric evaluation of a pediatric mucositis scale in cancer patients	For a pediatric mucositis scale in cancer patients ages 8 to 18, a Faces scale was found to be reliable, valid, and responsive.
Ng et al. 2012 [40]	Cross-sectional, observational study, Direct, A	VAS, NRS, and Faces scales	Investigate correlations between, and patient preference for, pain assessment scales for use in an oncology population	The VAS, NRS, and Faces scale showed a high degree of association with intensity of pain making these scales appropriate for pain assessment in cancer. The Faces scale was preferred over the VAS and NRS and was superior to the NRS or VAS with cognitively impaired patients
Chordas et al. 2013 [41]	Prospective observational study, Direct, A	11-point NRS, VAS, VRS	Determine if a single item pain measure can accurately identify clinically significant pain in a pediatric brain cancer population	In a pediatric population of brain cancer patients, a multi-item measure with VRS was more precise than a single item disease thermometer (variation of 11-point NRS).
Banthia et al. 2006 [42]	Prospective observational study, Direct, A	VAS and VRS	Comparison of daily versus weekly, unidimensional versus multidimensional measures of fatigue in a breast cancer population	A single item cancer fatigue VAS daily and weekly had some discordance between the daily and weekly measurement, indicating they are not capturing the same information. The single item fatigue VAS showed greatest overlap with the general fatigue subscale of the multidimensional fatigue measure, suggesting the VAS item is a unidimensional measure of one aspect of fatigue. The decision to use a multidimensional or unidimensional measures of fatigue will depend upon the research question.
Grassi et al. 2013 [43]	Cross-sectional, observational study, Indirect, C	NRS with Graphical component and multi-item measures	Validation and acceptance of the Distress Thermometer in an Italian cancer population	A distress thermometer (NRS with graphical component) was as specific and sensitive as multi-item measures and was slightly preferred by patients.

*VRS* verbal rating scale, *VAS* visual analogue scale, *NRS* numeric rating scale, *RA* rheumatoid arthritis, *PRO* patient-reported outcome

^a^Direct evidence: Primary research that compares different response scales within study. Indirect evidence: Review or expert opinion based on empirical evidence or primary research that evaluates a single response scale type within the study

^b^Grade Key: A) Primary research: compares different response scales within study; B) Review or expert opinion: based on an empirical evidence base; C) Primary research: evaluates a single response scale type within the study; and D) Review or expert opinion, based on expert consensus, convention, or historical evidence

#### Optimal number of response scale options

Literature on the optimal number of response scale options is presented in Table 4. In the comparison of a 5-point and 3-point VRS, there was evidence across studies that a 5-point scale was more informative and discriminative than a 3-point scale, but additional research was suggested [21]. Similarly, a 3-point scale was acceptable when compared to a 5-point scale if a simple scale was preferred based on the study population and construct of interest [22]. In a comparison of the 5-point VRS, 7-point VRS, and 11-point NRS scales to evaluate self-esteem, academic performance, and socioeconomic status, the 11-point NRS scale was more normally distributed than the shorter scale options, and demonstrated adequate validity; the authors therefore recommended selection of an 11-point NRS for self-reported measures used to assess social constructs [23]. An item response theory (IRT) analysis on the PROMIS items concluded that 4 to 6 was the optimal response set number; when more than 6 points were used, two or more response options were typically collapsed to improve model fit [24].

**Table 4:** Key studies that support response scale selection used in PRO instruments based on optimal response set number

Reference	Response Scale Type	Study Type, Evidence Type^a^, Grade^b^	Study Population	Summary of Results	Conclusion
Cleopas et al. 2006 [44]	Binary 3-point VRS 5-point VRS	Prospective study, Direct, A	1996 adult patients discharged from the hospital in Switzerland	Superior reliability, assessed by Cronbach’s alpha and test -retest, and convergent and discriminant validity for the 5-point version compared to the binary or 3-point version in the Nottingham Health Profile (NHP).	5-point VRS improved patient acceptability, reduced ceiling effects, and improved measurement properties
DeWalt et al. 2007 [24]	4-point VRS 5-point VRS 6-point VRS	Instrument development and/or validation study, Direct, A	Analysis of PROMIS items; pain, fatigue, emotional distress, physical function, and social function	Optimal response set number was somewhat dependent on the item and construct, 4 to 6 response options was typically optimal because this number both reduced cognitive burden for respondents and each option could provide unique information; investigators found that with response sets of greater than six choices, two or more options were typically collapsed to improve step-disorder and model fit.	Based on IRT analyses recommend 4-point to 6-point based on the item construct
Janssen et al. 2008 [45]	3-level 5-level	Instrument development and/or validation study, Direct, A	81 adult respondents in a panel session	5-level version had higher acceptability and comprehension and demonstrated superior reliability, validity, and discriminatory power.	5-level reduced ceiling effect, increased benefit in the detection of mild problems and in measuring general population health
Chomeya 2010 [46]	5-point Likert 6-point Likert	Instrument development and/or validation study, Direct, A	180 undergraduate students from Mahasarakham University	The 6-point Likert scale had slightly better discrimination and reliability, assessed by Cronbach’s alpha, compared to a 5-point scale.	Both the 5-point and 6-point scales gave discrimination at acceptable level per the standard of psychology tests
Rhodes et al. 2010 [47]	5-point Likert 7-point Likert	Instrument development and/or validation study, Direct, A	412 volunteer students in introduction psychology or physical education courses.	The 7-point scale (strongly disagree, moderately disagree, slightly disagree, undecided, slightly agree, moderately agree, strongly agree) had slightly higher reliability, assessed by Cronbach’s alpha, overall but predictive validity was largely comparable to the 5-point scale (strongly disagree, moderately disagree, undecided, agree, strongly agree). The 7-point scale demonstrated larger variability compared to the 5-point scale.	Either the 5-point or the 7-point scale is appropriate for use in scales for physical activity research
Bakshi et al. 2012 [22]	3-point Likert 5-point Likert	Instrument development and/or validation study, Direct, A	Inpatients aged 50 years and above in Singapore (*n* = 579); caregivers were interviewed as a patient proxy if the patient was not contactable, too weak, or had a language barrier.	The 3-point versions (disagree, neutral, and agree) were comparable to the 5-point versions (strongly disagree, disagree, neutral, agree, and strongly agree); the scores performed similarly. The 3-point versions were not less reliable, assessed by Cronbach’s alpha, or discriminative.	The 3-point scale is acceptable if a simple scale is required
Leung and Xu 2013 [23]	5-point VRS 7-point VRS 11-point NRS	Review, Indirect, B	7147 students (age 12 to 22 years) in Macau. 795 students in China. 844 secondary students in Macau.	Single item measures with an 11-point scale from 0 to 10 are closer to normality and interval scales, and have construct validity with major social constructs.	The 11-point scale was more normally distributed than the shorter scale options and had good validity.
Dumas et al. 2013 [21]	3-point VRS 5-point VRS	Review, Indirect, B	Published literature for the Scale to Assess Unawareness of Mental Disorder (SUMD).	The 5-point scale was more informative and discriminative than a 3-point scale.	Authors state that further research is required to determine if a 3-point or 5-point scale should be used with the SUMD.
Janssen et al. 2013 [48]	3-level 5-level	Instrument development and/or validation study, Direct, A	3919 adults with chronic conditions (cardiovascular disease, respiratory disease, depression, diabetes, liver disease, personality disorders, arthritis, and stroke)	For the 5-level system, the ceiling was reduced from 20.2% (3 L) to 16.0% (5 L). Absolute discriminatory power (Shannon index) improved considerably with 5 L (mean 1.87 for 5 L versus 1.24 for 3 L), and relative discriminatory power (Shannon Evenness index) improved slightly (mean 0.81 for 5 L versus 0.78 for 3 L). Convergent validity with WHO-5 was demonstrated and improved slightly with 5 L. Known-groups validity was confirmed for both 5 L and 3 L.	5-level version had higher acceptability and comprehension and demonstrated superior reliability, validity, and discriminatory power.

*PRO* patient-reported outcome, *VRS* verbal rating scale, *NRS* numeric rating scale

^a^Direct evidence: Primary research that compares different response scales within study. Indirect evidence: Review or expert opinion based on empirical evidence or primary research that evaluates a single response scale type within the study

^b^Grade Key: A) Primary research: compares different response scales within study; B) Review or expert opinion: based on an empirical evidence base; C) Primary research: evaluates a single response scale type within the study; and D) Review or expert opinion, based on expert consensus, convention, or historical evidence

## Discussion

The aim of this targeted literature review was to provide an overview of the response scale types commonly used in PRO instruments and to collate the empirical evidence for each type of scale. In the development of PRO instruments, the selection of the response scale(s) used should be based on the best available evidence.

Results for therapeutic area were limited based on the number of references provided for each disease state, thus, limiting the ability to recommend a type of response scale for a therapeutic area of interest. Empirical evidence suggests that a researcher’s choice of a VAS, NRS, VRS, or Faces scale is not based on the therapeutic area but on other aspects, such as study population (age), format of response option, and the concept being measured in the PRO instrument. The optimal number of response options depends on the construct and the number of items making up the domain of measure. A 5-point or 6-point VRS was more informative and discriminative than response scales with fewer response options, and that an 11-point NRS was more normally distributed than shorter scale options [21, 23]. However, while having more response options may be appropriate when assessing symptoms, it is important to consider the size of the instrument and the burden of response for patients, particularly if you are assessing functioning or daily activity, where such measures typically ask for a large set of responses. If these measures are being used as endpoints in a clinical trial setting, note that scores may vary depending not only on the overall number of items in the measure, but also the number of options for response to each individual item.

The intention of the literature review was to provide recommendations in the selection of response scale options for the development of new PRO instruments. But because the evidence is equivocal and there are several factors that needs to be taken into consideration, it is not as easy as providing broad recommendations. But we have provided a hypothetical case example to showcase value in collating the empirical evidence.

In this hypothetical example, a new PRO instrument needs to be developed to assess change in symptoms and change in functioning after patients are treated with a new compound as part of a clinical trial. There will be approximately 20 items and the evidence suggests that the VRS, NRS, and VAS are all appropriate response scale options for consideration.

- Selection: 6-point VRS

Justification: Empirical evidence suggest that data from an 11-point NRS was more normally distributed than a 5-point or 7-point VRS, but the developers decided to reduce the number of options given the larger number of items being asked of the subjects, therefore going with a VRS. Once the VRS and anchors were selected, the developers had to decide on the number of options, with evidence supporting anything between 4-points and 7-points. The objective was to select a scale that would discriminate between treatment arms; based on the evidence a 6-point scale showed slightly better discrimination and reliability compared to a 5-point scale and response sets of greater than 6 choices typically collapsed two or more options when scoring to improve model fit. This literature review was limited in that the key evidence was identified from articles published over the 10-year timespan from 2004 through 2014. Results were limited to a small number of studies that provided direct evidence, and multiple studies were difficult to compare given the variety in study design and diversity of terminology. The search strategy was based on pre-specified criteria that may not have been inclusive of global research using different terminology for PRO instruments. In the development of a PRO measure, the reliability, validity, and responsiveness is not only dependent on the response option, as examined in this study, but also on the item stem and concept being measured. The results of the literature review are limited to the evidence provided on only response scale variable and does not include investigation into how the psychometric properties are also related to the item stem.

Important considerations for response scale selection in PRO measures that were not addressed in the literature review include item response theory (IRT) and the use of Rasch analysis to support the type and format of response scales. IRT was not included as part of this literature review, since it was most likely not employed in older studies, which would mean there would be insufficient information to reach a valid conclusion. However, these types of analyses are now important in addressing the gaps in the literature to further assess the psychometric properties of items and their response options.

While the literature review identified an abundance of support for the VAS, this was based on historical data and does not take into consideration the preferences of patients or regulatory agencies when PRO instruments are used as primary or key secondary endpoints in clinical trials to support labeling claims. Further, this literature review did not demonstrate that the VAS was superior to other scale types in terms of psychometric properties or responsiveness. With the publication of the FDA Guidance in 2009 [25], PRO instrument development and selection of appropriate response scales for the context of use needs to be well documented, with evidence justifying the selection. Thus, when new instruments are being developed, it is important to elicit patient feedback regarding preferences and ease of use of different response scale types.

In summary, the VRS, NRS, and VAS, can all be acceptable response scale options in PRO instruments. However, when choosing a response scale type, it is important to consider the study objective and the context of use (i.e., construct being assessed, type of study population, frequency of assessment) during the development/modification of PRO instruments along with the study design.

## Abbreviations

FDA: Food and Drug Administration
IMMPACT: Initiative on Methods, Measurement, and Pain Assessment in Clinical Trials
NRS: Numeric rating scale
PRO: Patient-reported outcome
VAS: Visual analogue scale
VRS: Verbal rating scale
